# Muscle Fatigue Does Not Change the Effects on Lower Limbs Strength Caused by Aging and Parkinson’s Disease

**DOI:** 10.14336/AD.2018.0203

**Published:** 2018-12-04

**Authors:** Vinicius Alota Ignacio Pereira, Fabio Augusto Barbieri, Alessandro Moura Zagatto, Paulo Cezar Rocha dos Santos, Lucas Simieli, Ricardo Augusto Barbieri, Felipe Pivetta Carpes, Lilian Teresa Bucken Gobbi

**Affiliations:** ^1^Human Movement Research Laboratory (MOVI-LAB), Department of Physical Education, Sao Paulo State University (Unesp), Bauru, Brazil.; ^2^Posture and Gait Studies Laboratory (LEPLO), Department of Physical Education, Sao Paulo State University (Unesp), Rio Claro, Brazil.; ^3^Graduate Program in Physical Education and Sport at School of Physical Education and Sport of Ribeirao Preto (EEFERP), University of Sao Paulo, Centro Universitário Estacio de Ribeirao Preto, Brazil.; ^4^Applied Neuromechanics Group, Laboratory of Neuromechanics, Federal University of Pampa, Uruguaiana, Brazil.

**Keywords:** movement disorders, muscle strength, exhaustion, human movement

## Abstract

The aim of this study was to determine the impact of aging and Parkinson’s disease (PD) on lower limb muscle strength before and after muscle fatigue. One hundred thirty-five individuals were distributed over seven groups according to their age (20, 30, 40, 50, 60, 70 years old) and disease. Participants performed maximum voluntary isometric contractions (MVIC) in a leg press device followed by the muscle fatigue protocol (repeated sit-to-stand task). Immediately after muscle fatigue (less than 2 min), the MVIC were repeated. The peak force, peak rate of force development (first 50, 100, 200 ms), and root mean square and peak values of the vastus lateralis and vastus medialis muscle activity during MVIC were calculated before and after muscle fatigue. We found more pronounced reductions in lower limb muscle strength parameters (lower limb force, RFD-100 and RFD-200 - p<0.05) in individuals over 50 years of age and with PD. In addition, there was an inverse relation between aging and lower limb muscle strength parameters. The main findings were the lack of changes in peak force, RFDs and muscle activity of the vastus lateralis and vastus medialis after muscle fatigue according to aging and PD, and similar lower limb muscle strength parameters (before and after muscle fatigue) and effect of muscle fatigue in PD compared to the aged groups (60 and 70 years old groups).

One of the known effects of aging is muscle atrophy, which decreases muscle strength and power [1]. Muscle strength is known to reach peak values around the age of 35 [2] and declines by the sixth decade of life [3]. Furthermore, power, as measured by rate of force development (RFD), decreases with aging due not only to muscle changes but also alterations in passive components such as tendons [2], which may also impair postural stability [4] and torque production during walking [5]^.^ Reduced quadriceps muscle strength impairs gait speed [6] and step negotiation [7]. Such losses may be coupled with decreases in voluntary activation [8] and contribute to lower resistance to muscle fatigue in older people [9]. In addition, reduced lower limb strength is aggravated in people with Parkinson’s disease (PD), who present decreased isometric muscle strength compared to matched individuals [10,11]. Basal ganglia deficits are involved in the modulation of isometric force output [12], which is one of the potential causes of bradykinesia [13] and weakness [11]. In addition, people with PD present a robust system to adjust movements after lower limb muscle fatigue due to reduced lower limb muscle strength [11,14].

Muscle fatigue, as defined by any reduction in the force output of a muscle, caused by recent activation attributed to peripheral or central nervous system failure [15], reduces muscle strength in different ages and disease conditions (young people, older people, and people with PD) [6,11,16]. Fatigue also impairs RFD, a key contributor to age-related decrements in power and daily function [16,18]. Muscle fatigue has important implications for performance of daily life tasks as well as for safe locomotion, especially in older people and individuals with PD in conditions of perturbed gait and obstacle avoidance, possibly due to reduced balance control [14], that may increase the risk of falling [19]. Despite this, no study has analyzed the effects of muscle fatigue on lower limb muscle strength according to aging including individuals with movement disorders (PD), especially whether the effects of aging and PD on lower limb muscle fatigue are similar to unfatigued individuals.

The aim of this study, therefore, was to determine the impact of aging and PD on lower limb muscle strength parameters (lower limb force, RFD and muscle activity) before and after muscle fatigue. First, we determined when reduction in lower limb muscle strength parameters initiates due to aging and how PD influences this context. Second, we analyzed these effects on lower limb muscle strength parameters under the condition of muscle fatigue. We hypothesized that the reduction in lower limb muscle strength parameters (i.e. reduced lower limb force, RFD and muscle activity) would be more pronounced in individuals over 60 years of age and with PD. Moreover, we hypothesized that muscle fatigue would accelerate the reduction in lower limb muscle strength parameters (i.e., the effects would be more pronounced in individuals under 60 years of age). To test our hypothesis, we compared the lower limb force, RFD, and muscle activity of individuals of different ages and with PD, in a cross-sectional design, before and after inducing leg muscle fatigue.

## MATERIALS AND METHODS

### Participants

**Figure 1.**
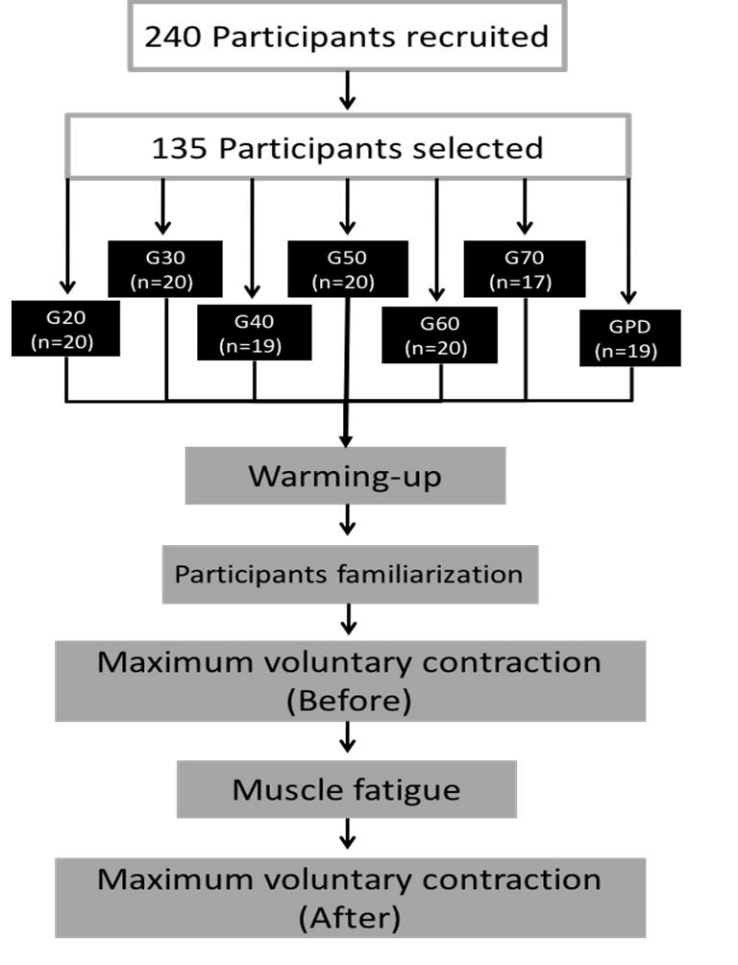
Flow diagram of the participants and experimental set-up.

Initially, two hundred and forty men from the local community were invited to participate in this study. To be included, they were required to be between twenty and eighty years old, living independently in the community; not present balance, bone, joint or muscle impairments that could limit execution of exercises; not present diabetes, obesity, hypertension, or elevated blood pressure at the time of the study (systolic >199 mm Hg or diastolic>109 mm Hg); have no history of heart attack, angioplasty, heart surgery, chest pain, shortness of breath, fainting or angina within the previous 3 months; no knee or ankle pain precluding testing; and for PD patients be in stage ≤ 3 on the Hoehn &Yahr Scale (H&Y) [20]. After applying these criteria, 135 individuals were included in the study (Figure 1). The volunteers were distributed over seven groups according to their age (20, 30, 40, 50, 60, and 70 years old) and disease (PD). The study was approved by the local Human Research Ethics Committee (#2055/2008), and all participants signed an informed consent form before joining the experiments.

### Experimental protocol

Participants were instructed to refrain from any strenuous physical activity for 48 h before the evaluations. When they arrived at the laboratory, the volunteers completed an anamnesis form to identify the exclusion criteria and questionnaires to determine physical activity level according to individual age - individuals aged up to 60 years old - habitual physical activity [21]; individuals over 60 years old - Modified Baecke Questionnaire for Older Adults [22] - and perception of fatigue (level of fatigue) -Multidimensional Fatigue Inventory (MFI) [23]. In addition, a neuropsychiatrist performed a clinical assessment of the patients with PD to determine the stage of the disease in each patient and perform an evaluation on the motor section of the Unified Parkinson’s Disease Rating Scale (UPDRS) [24].

Before the experiment, body mass and height were assessed. Body weight was measured with an electronic scale (precision 0.1 kg - Filizzola PL 150, Filizzola Ltda) and the height with a wall-mounted stadiometer (precision 0.1 cm - Sanny®, São Paulo, Brazil). Both measurements were carried out with participants wearing light clothing and no shoes. Body weight was measured with the participant positioned at the center of the weighing scale so that their weight was evenly distributed. All anthropometric measurements were performed by trained researchers, according to standardized techniques. Then, the participants performed a 5-min warm-up with stretching and movements in a leg press machine. In addition, they were familiarized with the maximum isometric voluntary force trials and anthropometric measurements were performed. Next, participants performed the maximum voluntary isometric contractions followed by the fatigue protocol. Immediately after fatigue (less than 2 min), the maximal voluntary contractions were repeated (Fig. 1).

### Lower limb maximum isometric strength

The lower limb maximum voluntary isometric contraction was performed in a leg press device attached to a force transducer (model 2000 NTM, EMG system TM, SP, Brazil) with sampling data at 2000 Hz and a precision of 0.98 N, which was used in combination with a signal amplifier (EMG System do Brazil Ltda.). The test was performed bilaterally with the volunteers requested to produce the maximum isometric force as fast as possible. An experimenter provided similar verbal encouragement to all participants. The participants were seated in a backward inclined chair, with the hip joint at 90º (180º is full extension) and knee joint at 110º (180º is full extension). Total contraction duration was 5 s. The average of two trials, with a 2-min rest between attempts, determined before and after muscle fatigue, was considered to determine lower limb force.

The raw force signals were filtered digitally by a fourth-order, zero-lag Butterworth low-pass filter at a cut-off frequency of 15 Hz. The onset of the contraction was defined as the point at which the force measured by the transducer exceeded 7.5 N above baseline [25]. The peak lower limb force was determined as the highest value registered within one-second from the onset of contraction (500-1500 ms) [26]. The peak rate of force development was determined by the steepest slope of the curve calculated with in windows of 25 ms (ΔForce/ΔTime), for the first 50, 100, and 200 ms (RFD-50, RFD-100 and RFD-200, respectively) after contraction onset [25].

Muscle activity was assessed using disposable Ag/AgCl surface-electrodes (lead-off area 1.0 cm^2^, inter-electrode distance 2.0 cm) in combination with a signal amplifier (EMG System do Brasil Ltda.). After abrasion and cleaning of the skin with alcohol, electrodes were attached over the vastus lateralis and vastus medialis from the right limb and following the fiber orientation. Electrode positioning and data acquisition followed the SENIAM guidelines [27]. Electrode position was carefully maintained before and after muscle fatigue induction. Electromyography signals were amplified (1000 times with common mode rejection ratio > 120 dB) and stored on a disc (12 bits AD converted, resolution ± 5 V, sample rate 1000 samples/s). Electromyography signals were band-pass filtered with a fourth-order, zero-lag, Butterworth filter with a cut off frequency between 20 and 500 Hz. Signals were then rectified and the root mean square (RMS) and peak values of electromyography calculated from the second that presented the least variation in the load-cell data, which included the peak lower limb isometric force. This one-second period was chosen by visual inspection and its location varied between the total five second period. The peak values of unfatigued trials were used to normalize the peak values of electromyography. The data acquisition systems were electronically synchronized.

### Muscle fatigue protocol

To induce lower limb muscle fatigue, participants performed a repeated sit-to-stand task from a standard chair (43 cm in height, 41 cm in width, and 42 cm in depth) without armrests. Participants were requested to keep their arms across the chest region during the exercise [6,7,14]. A metronome controlled the frequency of the sit-to-stand movement (30 cycles/min). The instructions given to the participants were: stand up in an upright position with knees fully extended, then sit back down and repeat the task at the beat of the metronome until you can no longer perform the task. The fatigue protocol was stopped when one of the following conditions was met: the participant indicated their inability to continue, the movement frequency dropped below and remained below 30 cycles/min after verbal encouragement, or after 30 min. The time to fatigue was recorded. The time between the fatigue protocol and the lower limb maximum voluntary contraction was as reduced as possible (<2 min), which was expected not to allow for full recovery [28]. The rating of perceived exertion was measured by the Borg Scale [29] at the beginning and end of the fatigue protocol.

### Statistical analysis

Statistical procedures were conducted using SPSS 15.0 for Windows?. The data were normally distributed, verified by the Shapiro-Wilk test. Effects of muscle fatigue were verified by paired Student’s t-tests for each group (α<0.05) to confirm that the individuals were fatigued. To identify group effects (G20, G30, G40, G50, G60, G70, and GPD) on participant characteristics, time to fatigue, rating of perceived exertion (initial and final), level of fatigue, and level of physical activity, one-way ANOVA was performed. Separate one-way ANOVAs were used for before and after muscle fatigue to identify group effects (G20, G30, G40, G50, G60, G70, and GPD) on lower limb muscle strength parameters (lower limb force, RFD-50, RFD-100, RFD-200, and RMS and peak values of vastus lateralis and vastus medialis). When the ANOVA indicated significant effects, Tukey's univariate tests were performed. The significance value was adjusted according to the number of comparisons (α<0.002). Effect sizes (Cohen's d) were calculated according to Cohen's convention [30]. The interpretation of effect sizes used was small (d<=0.2), medium (d<=0.5), and large (d<=0.8). In addition, the effects of PD on association of age and lower limb force parameters were explored in the partial Pearson correlation analysis with control for PD (α<0.05). Finally, the coefficients of variation, typical error and intraclass correlation for lower limb force between attempts before muscle fatigue were calculated according to Hopkins, Schabort and Hawley [31].

### RESULTS

Table 1 describes the participant characteristics, time to fatigue, rating of perceived exertion, level of physical activity, and level of fatigue for each group. Groups did not differ regarding body mass, rating of perceived exertion, and level of physical activity (p>0.05).

The GPD presented higher levels of fatigue (F_6,134_=6.73, p<0.001) than individuals over 50 years old (G50, G60 and G70 - p<0.001) and fatigued earlier (F_6,134_=7.22, p<0.001) than individuals under 40 years old (G20 and G30 - p<0.001). In addition, individuals over 70 years old and people with PD were shorter (F_6,134_=10.99, p<0.001) than individuals under 50 years old (G20, G30 and G40 - p<0.001).

**Table 1:** Means and standard deviations of participant characteristics, body mass index (BMI), time to fatigue, rating of perceived exertion (initial and final, with range of values between parentheses), level of physical activity and level of fatigue for each group. In addition, H&Y and UPDRS-motor values for people with PD are presented.

		G20 (n=20)	G30 (n=20)	G40 (n=19)	G50 (n=20)	G60 (n=20)	G70 (n=20)	GPD (n=19)
Age (years)		24 ± 3	32 ± 3	44 ± 3	54 ± 3	64 ± 3	75 ± 4	68 ± 9
Body mass (kg)		77.96±14.74	79.26 ± 14.23	84.74 ± 15.93	82.17 ± 13.40	77.99 ± 12.61	72.45 ± 12.40	72.97 ± 9.10
Height (m)		1.78 ± 0.04	1.74 ± 0.06	1.74 ± 0.06	1.71 ± 0.07	1.71 ± 0.06	1.65 ± 0.06	1.65 ± 0.07
BMI (score)		24.36 ± 4.26	26.02 ± 3.98	27.45 ± 4.31	27.86 ± 4.32	26.55 ± 3.65	26.51 ± 3.29	26.66 ± 2.64
Time to fatigue (min)		12.00 ± 10.50	12.90 ± 11.00	8.78 ± 10.06	6.50 ± 7.00	4.8 ± 3.43	3.09 ± 2.51	1.53 ± 0.82
Rating of perceived exertion (scale)	Initial	7.35 ± 1.42	6.85 ± 1.35	7.95 ± 2.01	7.30 ± 1.72	8.45 ± 2.24	8.82±1.19	8.16 ± 1.80
		(6 - 11)	(6 - 11)	(6 - 13)	(6 - 11)	(6-13)	(7-11)	(6 - 11)
	Final	19.15 ± 1.14	18.70 ± 1.42	18.58 ± 1.35	19.32 ± 0.82	18.60 ± 1.60	17.53±1.55	17.21 ± 2.70
		(17 - 20)	(16 - 20)	(15 - 20)	(17 - 20)	(15-20)	(15-20)	(13 - 20)
Level of physical activity (score)		8.15 ± 2.67	7.98 ± 1.89	7.93 ± 1.78	7.11 ± 8.56	5.82 ± 5.61	6.88±6.63	4.50 ± 3.18
Level of fatigue (score)		47.20 ± 8.08	45.70 ± 8.60	43.84 ± 9.85	39.05 ± 11.23	37.45±9.43	36.71±6.34	53.42 ± 14.97
							H&Y (score)	1.79 ± 0.35 (1 - 2.5)
							UPDRS-motor (score)	34.32 ± 8.47 (10 - 48)

### Effects of muscle fatigue protocol on lower limb muscle strength parameters

Coefficients of variation (7.07%), typical error (209.73N) and intraclass correlation (0.98) enhanced the reproducibility and confirmed the reliability of lower limb maximum isometric strength.

**Figure 2.**
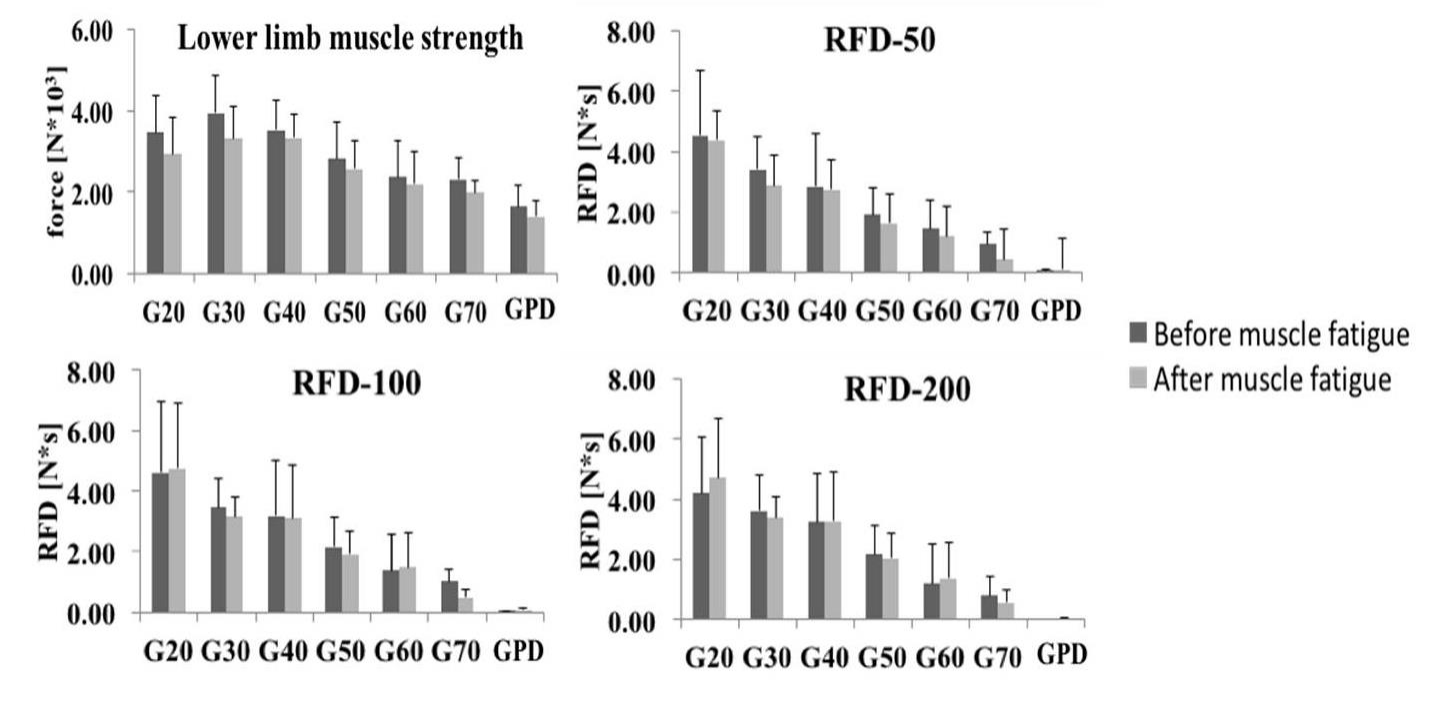
Means and standard deviations of lower limb strength parameters (lower limb muscle force and rate of force development in 50, 100, and 200 ms - RFD-50, RFD-100, RFD-200) before and after muscle fatigue for each group.

All groups presented reduced lower limb force after muscle fatigue (G20 - t_19_=4.18, p<0.001 - reduction of 15.36%; G30 - t_19_=5.69, p<0.0001 - reduction of 15.74%; G40 - t_19_=2.22, p<0.05 - reduction of 5.58%; G50 - t_19_=3.33, p<0.004 - reduction of 8.92%; G60 - t_19_=3.33, p<0.004 - reduction of 7.39%; G70 - t_19_=4.66, p<0.001 - reduction of 13.44%; GPD - t_19_=2.44, p<0.02 - reduction of 14.93%; Figure 2 and Table 2 - moderate effect size: from 0.20 to 0.71). In addition, muscle fatigue reduced the RFD-50 and RFD-100 in individuals over 70 years old (t_16_=5.03, p<0.0001 - large effect size: 1.71; and t_19_=4.19, p<0.001 - large effect size: 1.53, respectively) and the RFD-100 in people with PD (t_19_=-2.54, p<0.02 - moderate effect size: 0.72). Individuals of the G20 (t_19_=3.84, p<0.001 - large effect size: 1.23), G50 (t_19_=8.62, p<0.0001 - large effect size: 2.34), G70 (t_16_=8.41, p<0.0001 - large effect size: 3.12), and GPD (t_18_=7.81, p<0.0001 - large effect size: 3.10) demonstrated reduced peak value of the vastus lateralis while the G30 (t_19_=2.10, p<0.05 - large effect size: 1.13), G40 (t_18_=2.50, p<0.02 - moderate effect size: 0.70) and G70 (t_19_=4.79, p<0.0001 - large effect size: 3.24) presented reduced RMS of the vastus medialis after muscle fatigue. Finally, the G20 (t_19_=-4.16, p<0.001 - large effect size: 1.04) and G40 (t_18_=-2.24, p<0.03 - moderate effect size: 0.51) groups showed increased RMS of the vastus lateralis after muscle fatigue while the G50 presented a reduced value (t_19_=2.17, p<0.04 - moderate effect size: 0.55).

**Table 2:** Summary of the effects of aging and PD on lower limb muscle strength parameters before and after muscle fatigue and its effects size.

	Effects of aging and PD - without muscle fatigue	Effects of aging and PD - under muscle fatigue
Lower limb force	G20, G30, G40>G50, G60, G70, GPD - large effect size: from 0.86 to 2.98 G50>GPD - large effect size: 1.65	G20>G70, GPD - large effect size: 1.38 and 2.18, respectively G30, G40>G60, G70, GPD - large effect size: from 1.41 to 3.84 G30, G50>GPD - large effect size: 3.07 to 2.15, respectively

RFD-50	G20>G30, G40, G50, G60, G70, GPD - moderate to large effect size: from 0.67 to 2.94 G40>G70, GPD - large effect size: 1.54 and 2.29, respectively G50>GPD - large effect size: 2.80	G20, G30, G40>G50, G60, G70, GPD - large effect size: from 0.86 to 2.73 G30, G40>G60, G70, GPD - large effect size: from 1.20 to 5.95 G30, G50>GPD - large effect size: 5.95 to 2.81, respectively

RFD-100	G20, G30, G40>G50, G60, G70, GPD - large effect size: from 1.18 to 5.05 G50>GPD - large effect size: 3.21	G20, G30, G40>G50, G60, G70, GPD - large effect size: from 0.83 to 3.06 G30, G40>G60, G70, GPD - large effect size: from 1.80 to 6.79 G30, G50>GPD - large effect size: 6.79 to 3.46, respectively

RFD-200	G20, G30, G40>G50, G60, G70, GPD - large effect size: from 0.81 to 2.80 G50>GPD - large effect size: 2.44	G30, G40>G60, G70, GPD - large effect size: from 1.32 to 6.89 G20>G50, G60, G70, GPD - large effect size: from 1.80 to 3.40 G30, G50>GPD - large effect size: 6.89 to 3.52, respectively

RMS VL	G20>G30, G40, G50, G60, G70, GPD - large effect size: from 0.88 to 1.71 G40>GPD - large effect size: 1.55	G20>G30, G40, G50, G60, G70, GPD - large effect size: from 1.69 to 3.26 G30>G50, G60, G70, GPD - large effect size: from 1.17 to 1.50

RMS VM	G20>G30, G40, G50, G60, G70, GPD - large effect size: from 1.69 to 2.42	G20>G30, G40, G50, G60, G70, GPD - large effect size: from 1.63 to 2.35

Peak VL	No effects	G30, G50>GPD - large effect size: 1.45 to 1.20, respectively

Peak VM	No effects	No effects

VL - vastus lateralis; VM - vastus medialis; Peak - Peak value of electromyography.

### Effects of aging and PD on lower limb muscle strength parameters

Individuals under 30 years old (G20) presented higher RFD-50 (F_6,134_=29.28, p<0.0001) and RMS of the vastus lateralis (F_6,134_=16.05, p<0.0001) and vastus medialis (F_6,134_=25.13, p<0.0001) than the other groups (G30, G40, G50, G60, G70, and GPD - p<0.001) (Figure 2 and Table 3). Individuals under 40 years old presented higher lower limb force (F_6,134_=20.04, p<0.0001), RFD-100 (F_6,134_=26.56, p<0.0001), and RFD-200 (F_6,134_=31.03, p<0.0001) than individuals over 50 years old and with PD (G50, G60, G70 and GPD - p<0.001). In addition, individuals over 60 years old and with PD presented lower limb force than the G40 group (p<0.001), RFD-100 and RFD-200 compared to the G30 and G40 groups (p<0.001), and RFD-50 compared to the G30 group (p<0.001). Older individuals (G70) and people with PD demonstrated decreased RFD-50 compared to the G40 group (p<0.001). Finally, people with PD presented reduced lower limb force, RFD-50, RFD-100, and RFD-200 than the G50 group (p<0.001) and RMS of the vastus lateralis than the G40 group (p<0.001).

**Figure 3.**
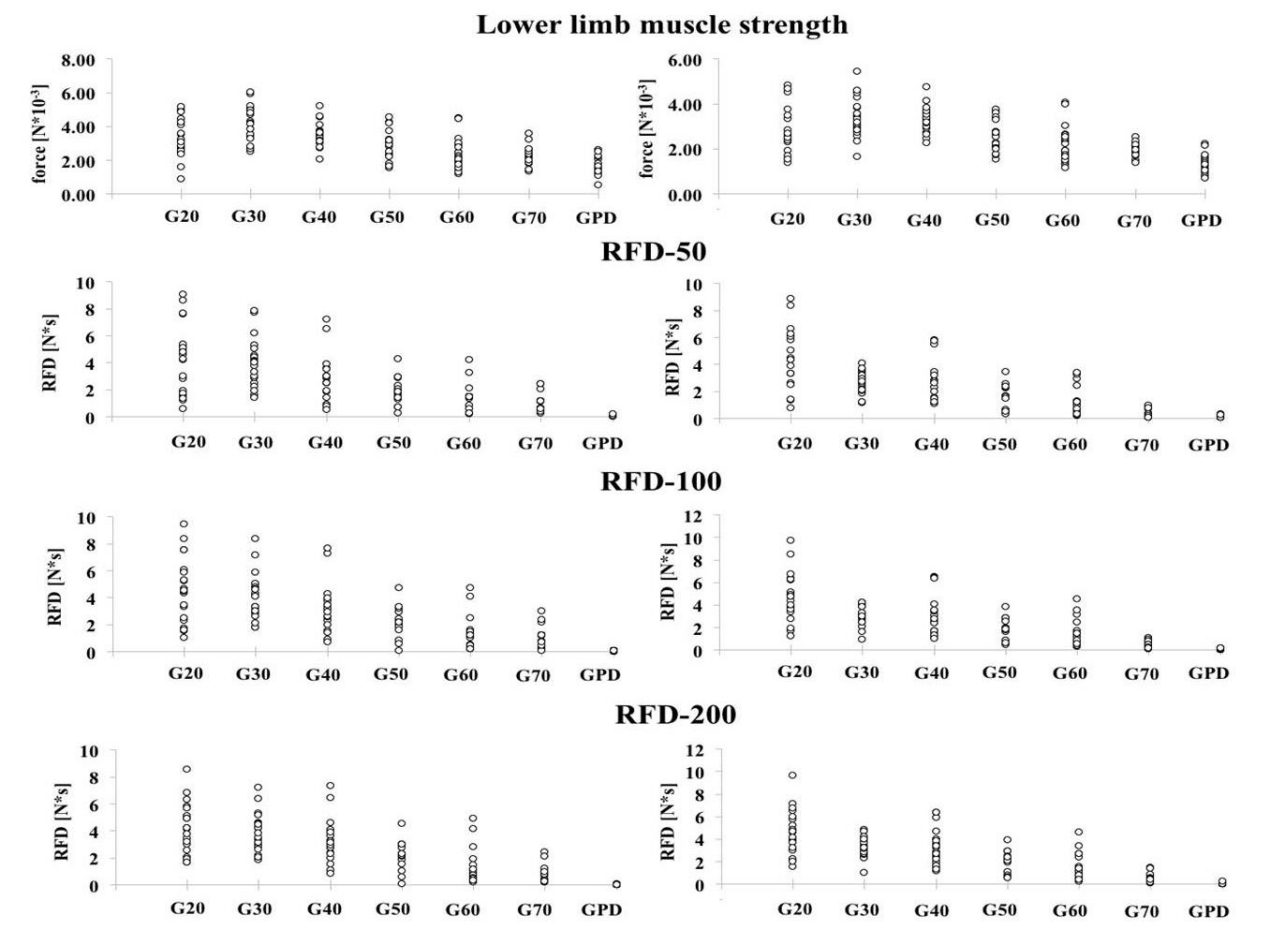
Association between aging, PD, and lower limb muscle strength parameters before (left panel) and after (right panel) muscle fatigue.

There was a moderate inverse correlation between age and lower limb muscle strength parameters before muscle fatigue, supporting the deficits in muscle strength reported due to age advances (lower limb force: r=-0.46, p<0.0001; RFD-50: r=-0.60, p<0.0001; RFD-100: r=-0.62, p<0.0001; RFD-200: r=-0.68, p<0.0001) (Fig. 3).

### Impact of aging and PD on muscle strength in a fatigue state 

As in the unfatigued test, after muscle fatigue individuals under 30 years old (G20) presented higher RFD-50 (F_6,134_=32.62, p<0.0001), and RMS of the vastus lateralis (F_6,134_=52.30, p<0.0001) and vastus medialis (F_6,134_=31.88, p<0.0001) than the other groups (G30, G40, G50, G60, G70, and GPD - p<0.001), however, the RFD-100 (F_6,134_=35.33, p<0.0001) was also higher for the G20 group compared to the other groups (Figure 2 and Table 3). Besides, G20 group only presented higher lower limb force (F_6,134_=22.39, p<0.0001) than individuals over 70 years old and with PD (p<0.001) after muscle fatigue. In addition, the G30 and G40 groups presented higher lower limb force and all RFDs (50, 100 and 200 - F_6,134_=40.61, p<0.0001) than individuals over 60 years old and with PD (p<0.001). Moreover, individuals over 50 years old presented lower RFD-200 than the G20 group (p<0.001) and RMS of the vastus lateralis than the G30 group (p<0.001). Finally, the G30 and G50 groups presented higher lower limb force, RFD-50, RFD-100, and RFD-200, and peak value of the vastus lateralis (F_6,134_=5.63, p<0.0001), respectively, than people with PD (p<0.001).

There was a moderate inverse correlation between age and lower limb muscle strength parameters after muscle fatigue (lower limb force: r=-0.45, p<0.0001; RFD-50: r=-0.68, p<0.0001; RFD-100: r=-0.68, p<0.0001; RFD-200: r=-0.72, p<0.0001), as before muscle fatigue (Fig. 3).

## DISCUSSION

In the present study, we investigated when a reduction in lower limb muscle strength was initiated due to aging and how PD influences this context. This seems to be the first study to analyze the effects of aging on lower limb muscle strength parameters (lower limb force, RFD and muscle activity) including comparison of groups with and without movement disorders. Previous studies have described the deleterious effects of muscle fatigue on muscular performance [6,11,16], which motivated us to analyze lower limb muscle strength in individuals with and without movement disorders when facing a fatigue condition. We hypothesized that age over 60 years and PD would elicit impairment in lower limb muscle strength parameters, especially under a fatigue state. Our hypothesis was partially confirmed. We found more pronounced reduction in lower limb muscle strength parameters (lower limb force, RFD-100 and RFD-200) over 50 years of age and with PD. In addition, higher lower limb force, RFDs and muscle activity were observed in individuals aged 20 to 39 years old, and there was an inverse relation between aging and lower limb force and RFD. The main novelties in our study were the lack of changes in peak force, RFDs and muscle activity of the vastus lateralis and vastus medialis after muscle fatigue according to aging and PD, which was contrary to our second hypothesis (muscle fatigue would accelerate the effects on lower limb muscle strength parameters in aging and PD), and a very interesting similar lower limb muscle strength parameters (before and after muscle fatigue) and effect of muscle fatigue in the PD compared to the aged groups (60 and 70 years old groups).

The reduction in lower limb muscle strength parameters (lower limb force, RFD and muscle activity) in the aged groups did not differ under a fatigue state. Our findings are the first to demonstrate that effects of aging on reduction in lower limb muscle strength parameters are independent of muscle fatigue. This is an interesting finding as muscle fatigue is characterized by reductions in muscle strength in different ages and disease conditions [6,11,14,32], and impairs neuromuscular function [16,18], the peripheral proprioceptive system, and the central processing of sensory inputs [33]. We can explain the lack of a fatigue effect on lower limb muscle strength parameters during aging by 1) controversial effects of fatigue in aging: substantial literature suggests an advantage in terms of fatigue resistance on the part of older muscle [34,35], but this is not always observed. Several studies suggest that there is no difference in fatigue between young and old individuals [36,37], and some reports have observed more fatigue in the elderly [9]. The discrepant results across studies could be attributable to differences in contraction mode, testing protocol, muscle group or subjects’ characteristics; 2) protocol to fatigue: the effects of fatigue are exercise-dependent [38]. We conducted a protocol that requires muscle resistance. Older adults may be more susceptible to fatigue during high-velocity dynamic contractions [39]; 3) robustness of aging process: aging is a robust process that involves many components and body systems. The effects of muscle fatigue in this process is still controversy; 4) age-related changes in muscle fiber characteristics: aging causes changes in muscle mass, innervation properties and contractile apparatus, which can change the effects of muscle fatigue on lower limb muscle strength parameters. Thus, our results may suggest that the reduction in lower limb muscle strength parameters in aging was not muscle fatigue-dependent, which seems to be related to a reduction in muscle strength.

People with PD demonstrated no reduction in lower limb force, RFDs (50, 100 and 200 ms) and muscle activity (RMS and peak values of vastus lateralis and medialis) before and after muscle fatigue compared to individuals over 60 years old. This is surprising since PD is known to reduce muscle strength [10,11] and lower absolute and relative RFD to approximately half the capacity of neurologically health peers [40]. In addition, people with PD present a strong and negative correlation of central activation deficits with quadriceps strength, suggesting that this may be an important mechanism driving strength loss [11]. However, our group with PD was at an initial stage of the disease (H&Y - 1 to 2.5 and UPDRS motor - 20 to 48 pts), which could explain the lack of increased effects in lower limb muscle strength parameters. Despite this, reduction in lower limb muscle strength parameters has been presented in previous studies for this population [10,11,14]. The finding is even more interesting after muscle fatigue since individuals with PD have a mitochondrial dysfunction that contributes to muscle fatigue [41]. Our results suggest that impairments in motor control and degradation in sensorimotor integration and sensory feedback presented by people with PD [42] have no effects on the role of muscle fatigue and lower limb muscle strength parameters. However, we need to consider the reduced time that the people with PD performed the muscle fatigue protocol, which could have been caused by exacerbated fatigue perception of people with PD [43] and maybe insufficient to cause muscle overload and induce metabolic fatigue [11], even with a reduction in muscle strength. Therefore, the interesting findings regarding lower limb muscle strength and effects of muscle fatigue seem to be important for prescription and monitoring of physical exercise for this population.

**Table 3:** Means and standard deviations of vastus lateralis and vastus medialis muscle activity (RMS and peak value) before (BMF) and after (AMF) muscle fatigue for each group.

	G20		G30		G40		G50		G60		G70		GPD	
	BMF	AMF	BMF	AMF	BMF	AMF	BMF	AMF	BMF	AMF	BMF	AMF	BMF	AMF
RMS vastus lateralis	184.10 (135.36)	323.96 (132.80)	90.88 (65.62)	124.77 (102.53)	44.62 (19.06)	57.05 (29.01)	53.01 (29.28)	38.42 (23.49)	43.90 (29.80)	35.96 (11.16)	43.32 (20.41)	42.74 (19.80)	19.96 (11.88)	16.88 (8.96)
RMS vastus medialis	191.59 (102.14)	187.29 (102.68)	64.58 (30.01)	47.29 (22.95)	85.72 (71.79)	58.14 (44.39)	43.68 (36.94)	35.02 (27.54)	25.66 (15.32)	29.79 (18.84)	50.05 (28.73)	29.80 (18.56)	15.62 (10.90)	16.29 (9.53)
Peak value of vastus lateralis (%)	96.97 (4.45)	82.14 (16.48)	95.86 (4.46)	88.16 (17.46)	92.90 (5.50)	83.89 (23.67)	95.27 (6.05)	70.14 (13.92)	91.29 (5.73)	86.18 (14.33)	94.91 (5.82)	72.03 (8.58)	95.11 (6.76)	67.11 (10.84)
Peak value of vastus medialis (%)	97.75 (8.05)	76.46 (21.53)	93.66 (5.58)	75.90 (21.52)	95.72 (4.13)	88.39 (14.15)	96.37 (5.78)	71.13 (20.68)	95.83 (4.28)	83.74 (23.84)	94.09 (5.63)	59.04 (14.21)	95.98 (5.56)	82.81 (12.14)

Aging was related to reduction in lower limb muscle strength parameters (lower limb force, RFD and muscle activity), which was more pronounced after 50 years of age. Previous studies have already well established the effects of aging on lower limb muscle strength, such as a reduction in muscle mass and size, loss of individual fibers, especially type II fibers, and a decrease in explosive strength characteristics of the neuromuscular system [44]. In this regard, our paper adds that the age of the critical threshold of reduction in lower limb muscle strength was over 50 years of age. The fifth decade of life is characterized by a decrease in whole muscle size (about 30% from 50 years old to 80 years old) [45] and fiber count (about 35% from 50 years old to 75 years old) [45,46]. This translates to an annual decrease of 1% in whole muscle cross-sectional area beyond the fifth decade of life [44], and a strength decrease of nearly 15% per decade [32]. In addition, possibly after 50 years old there is a reduction in the ability of the central nervous system to activate the agonist muscles [47] due to the reorganization of areas in the brain and remodeling of motor units [48], causing effects on muscle strength. The preoccupation with the reduction in lower limb muscle strength precedes the sixth decade of life, which was indicated as the period when significant reduction in muscle strength begins [3]. Furthermore, decreased RFD after 50 years old indicated a reduction in the ability to generate force quickly, which may be more functionally relevant than the ability to generate maximum lower limb force. The activities of daily living, such as locomotion, are characterized by limited strength development time (50-200 ms), which is considerably smaller than necessary to reach the maximum force (≥ 300 ms). Preservation of lower limb muscle strength and the capacity to generate force quickly are important due to decreased in these parameters leading to mobility disability, contributing to age-related declines in gait speed, and being consistently associated with mortality across populations [49]. Therefore, we can suggest that the effects of aging on lower limb muscle strength parameters occurred earlier than expected and exercise training should consider our findings. In the present study, we opted to analyze strength of a muscular group that plays a fundamental role in locomotion, however we should keep in mind that the effects of aging on muscle strength are muscle-dependent [50].

We concluded that muscle fatigue did not influence the effects of aging in the reduction in lower limb force, RFD and muscle activity. In addition, the effects on lower limb muscle strength parameters were more pronounced after 50 years of age. Our findings indicated that there is a relationship between reduction in lower limb force and RFD, and aging. Finally, PD had no effects on lower limb muscle strength parameters compared to neurologically healthy individuals over 60 years of age, which seems to indicate that the reduction in lower limb muscle strength was caused specifically by aging.

Our findings reinforce the preoccupation with reduction on muscle strength parameters after 50 years old. It is worrisome due to changes in muscle strength parameters is closely linked to mobility disability and the incidence of accidental falls, which typically result in further health complications. However, lower limb muscle strength may be trained, independently the effects of muscle fatigue, to avoid reduction in the quality of locomotion. In addition, people with PD presents similar capacity of produce lower limb strength compared to neurologically healthy individuals over 60 years of age. This is an important implication since previous studies indicated lower limb strength loss with PD due to central activation deficits [10,11]. Therefore, future studies are necessary to help in the explanation about the effects aging and PD disease in lower limb strength loss in this population.

Although the findings shed some light on the impact of aging and PD on lower limb muscle strength parameters before and after muscle fatigue, some limitations are evident. First, the differences between groups for time to fatigue and level of fatigue, specially for people with PD, may be influenced the lower limb muscle strength parameters. However, the relationship between these parameters and the time to fatigue and level of fatigue was not identified. In addition, people with PD commonly report more fatigue symptoms compared to neurologically healthy individuals [43]. Besides, time to fatigue is highly related to aging [1,6], which is difficult to control during the experiment. Second, our findings were related to lower limb, which is difficult to consider for other muscle region, such as upper limb. Considering these limitations, results should be done with care.
